# Multi-Scale Stress Wave Simulation for Aggregates Segregation Detection of Concrete Core in Circular CFST Coupled with PZT Patches

**DOI:** 10.3390/ma11071223

**Published:** 2018-07-17

**Authors:** Hongbing Chen, Bin Xu, Yilung Mo, Tianmin Zhou

**Affiliations:** 1 College of Civil Engineering, Hunan University, Changsha 410082, China; chb@hnu.edu.cn; 2 College of Civil Engineering, Huaqiao University, Xiamen 361021, China; 3 Department of Civil and Environmental Engineering, University of Houston, Houston, TX 77204-4006, USA; ymo@uh.edu (Y.M.); tzhou@uh.edu (T.Z.)

**Keywords:** piezoelectric lead zirconate titanate (PZT), circular concrete-filled steel tubular (CCFST), random aggregates method, multi-scale simulations, numerical concrete, aggregate segregation detection, wavelet packet analysis

## Abstract

In this study, the numerical investigation of the detectability of concrete aggregate segregation in circular concrete-filled steel tubulars (CCFST) based on piezoelectric lead zirconate titanate (PZT) measurement is performed. The stress wave propagation in the concrete core of circular CCFST excited with a surface-mounted PZT actuator is studied with multi-scale and multi-physical field coupling analysis. The piezoelectric effect of PZT patches and its coupling effect with CFSTs are considered. Numerical concrete modeling technology is employed to construct the concrete core composed of randomly distributed aggregates with and without aggregate segregation at different levels, mortar, and an interfacial transition zone (ITZ). The effects of the random distribution of elliptical aggregates, aggregate segregation, and the existence of ITZ in the concrete core on the wave fields in the cross-section and the corresponding voltage response of the embedded PZT sensor are discussed. An evaluation index based on wavelet packet analysis on the output voltage response is defined, and its sensitivity to concrete aggregate segregation is systematically investigated. The multi-scale and multi-physics coupling simulation results indicate that concrete aggregate segregation in the concrete core of CFST members can be efficiently detected based on the stress wave measurement with a PZT sensor.

## 1. Introduction

Concrete-filled steel tubular (CFST) members present excellent performance with high load-carrying capability, stiffness, and good ductility under strong dynamic excitations and earthquakes, as well as remarkable economic benefits in construction. Therefore, CFST components have been widely employed in high-rise buildings, long-span bridges, metro stations, industrial plants, and harbor engineering structures. Besides the general rectangular and circular CFST columns with large cross-sections, multi-chamber CFST members composed of several irregular polygonal cross-sections have been widely adopted as vertical load-carrying components in skyscrapers. For example, multi-chamber CFST columns with a cross-section area of about 45 m^2^ have been used at the foot of a super high-rise building with a design height of 597 m in China, which represents the largest multi-chamber CFST column reported till now [1]. Usually, the concrete used for the construction of CFST members is the self-consolidating concrete (SCC) due to its convenience in construction. The workability parameters including the filling ability (flowability), passing ability, and stability (aggregate segregation resistance) of SCC are critical, and should be assessed in the initial mixture design. The separation resistance to keep the distribution of coarse and fine aggregates uniform is one of the most important requirements for SCC [2,3].

Conceptually, an unreasonable proportion mix design with poorly graded aggregates and excessive water content leads to the settlement of aggregates under the effect of self-weight. During the construction of CFST structures in high-rise buildings, SCC is usually cast every two or three stories with a dropping height of several meters, and concrete aggregate segregation is one of the most concerned issues in practice. The complicated internal structures, including horizontal diaphragm plates, vertical stiffening ribs, and heavy reinforcement in the concrete core of large-scale CFST members might lead to difficulties in ensuring a uniform distribution of aggregates, and the heavier coarse aggregates most likely settle down and concentrate on the bottom of CFST members.

Numerical analysis on the structural behavior of concrete structures can be performed at the macro level (three to four times the maximum aggregate volume), the meso level (10^−4^–10 cm), and the micro level (10^−7^–10^−4^ cm), as reported by Chen et al. [4]. It has been pointed out that the distribution variation of aggregates significantly affect the structural performance of concrete specimens subjected to uniaxial loading due to the material difference between that of the aggregates, the interfacial transition zone (ITZ), and mortar. Moreover, the failure processes of modeled recycled aggregate concrete under uniaxial compression are affected by the lack or variation of circular aggregates [5]. Moreover, Classen opined that the pry-out resistance of open rib shear connectors in cracked concrete was influenced by the aggregate interlock mechanism, which controlled the transfer of shear stresses across the crack [6]. In essence, the aggregate interlock directly depends on the distribution pattern of aggregates at the meso level, resulting in different shear capacities. The aggregate segregation of the concrete core in CFST members easily leads to a decrease in concrete strength due to the non-uniform material distribution at the meso level, and finally causes a decrease in the mechanical performance of CFST members, including load-carrying capacity, ductility, and long-term properties.

Checking the homogeneity of the concrete core in CFST members is essential to guarantee the expected mechanical behavior of the concrete core and CFSTs. Real-time, robust, and inexpensive non-destructive testing (NDT) technologies are critical for the aggregate segregation and integrity inspection of concrete structures. Even though several NDT technologies are available for the damage detection and condition monitoring for reinforced concrete (RC) structures and concrete–steel composite structures, efficient concrete aggregate segregation detection for CFST members is still a challenging task [7]. Even though some NDT techniques, such as the ultrasonic method, acoustic emission, infrared thermography, electromagnetic method, X-ray, and radar/microwave approaches, have been widely employed in structural health monitoring (SHM), no efficient methods have been proposed for the inspection and monitoring of the concrete aggregate segregation in CFST members [8,9,10,11,12,13]. Until now, available on-site concrete aggregate segregation assessment methods are extremely limited [14]. For aggregate segregation detection in concrete members, a simple, low-cost near-field microwave non-destructive inspection technique has been reported, and the results indicated that the standard deviation of the magnitude of the reflection coefficient measurement was linearly correlated with the aggregate density in concrete [15]. Breul et al. presented a geoendoscopy and automatic image processing technique for concrete homogeneity measurement and particle size distribution control [16]. Compared with SCC, the practical inspection of the aggregate segregation in CFST members is much more challenging because of the inaccessibility of the concrete core confined with steel tubular. A PZT-based ultrasonic time-of-flight (TOF) method has been adopted to assess the concrete infill condition of concrete-filled, fiber-reinforced polymer tubulars [17]. The electromagnetic method (EM) can be employed to detect the interface debonding for fiber-reinforced polymer (FRP)-jacketed concrete structures. However, it is not suitable for the debonding detection of CFST members, since the shielding effect of steel tubular, which makes the penetration of an electromagnetic wave through the metallic media impossible [18]. Therefore, it is critical to find a novel and efficient approach to detect the concrete segregation in CFST members.

Due to distinct advantages including low-cost, fast response, long life service, and good linearity properties, piezoceramic lead zirconate titanate (PZT) patches have been employed as actuators or sensors in the SHM, and damage detection for various civil engineering structures. In recent years, aiming at the interface debonding detection for CFST members, Xu et al. proposed a PZT-based active interface debonding detection approach for CFST members. The stress wave measurement propagating within the cross-section of CFST has been studied, and the proposed approach has been used for the condition evaluation of CFST members in high-rise buildings [8,9]. Experimental observations showed that the defined evaluation indices based on wavelet packet energy or the wavelet packet energy spectrum were sensitive to the interface debonding defect [8,9]. Recently, Xu et al. performed numerical studies on the stress wave propagation characteristics in both circular and rectangular CFST members where the concrete core is simulated with homogeneous material assumption [10,11,12,13].

In order to investigate the effect of the meso-scale structure and the randomness of aggregates distribution in the concrete core of CFST members on the stress wave propagation and the response of PZT sensors, multi-scale simulation has been carried out to distinguish the dominance of debonding defects by using the numerical concrete modeling technology where the aggregates shapes, distributions, and the interface between aggregates and mortar can be considered [19]. Moreover, the meso-scale modeling approaches have also been employed to investigate and explain the stochastic behavior of the concrete structure. The lattice model [19], random particle model [20,21,22], random aggregate model [23,24], stochastic mechanical characteristic model [25,26], and so on have already been proposed and widely adopted in meso-scale simulation for concrete structures. Among these numerical models, the random aggregate model (RAM) has been recognized as one of the most powerful approaches, which discretizes concrete as the mixture of coarse and fine aggregates, mortar, the interfacial transition zone (ITZ), and initial pores at the meso scale. As it is difficult to experimentally mimic the aggregates segregation of the concrete core in CFST, the multi-scale modeling technology for concrete, which can establish aggregates with different geometrical shape and distribution patterns, can provide a powerful approach to simulate the aggregate segregation in the concrete core of CFST members.

In this study, with the help of the multi-scale modeling approach of RAM, multi-scale and multi-physical field coupling numerical simulation on the stress wave propagation within the cross-section of CFST members with and without aggregate segregation is carried out. In addition to the piezoelectric effect of PZT patches, the coupling effects between the surface-mounted PZT actuator and steel tubular, as well as the coupling effect between the embedded PZT sensor and concrete core are considered. The effects of the random distribution of elliptical aggregates, concrete segregation, and the interfacial transition zone (ITZ) on the voltage response of the embedded PZT sensor in the concrete core of CFST members excited by a PZT actuator under sweep frequency signal are investigated. The numerical findings indicate that the signal amplitude of output voltage obtained from the embedded PZT sensor and the traveling time are affected by the aggregates segregation in the concrete core. An evaluation index is defined based on the wavelet packet analysis on the embedded PZT sensor measurement, and its relationship with the severity of concrete segregation is also investigated.

## 2. Multi-Physical and Multi-Scale PZT–CFST Coupling Model with Numerical Concrete Core

**Figure 1:**
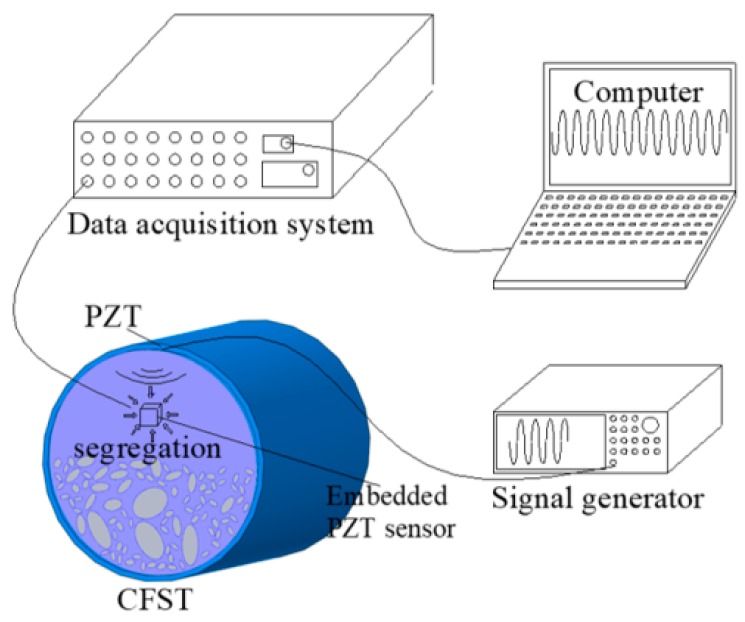
Aggregate segregation detection method for concrete core in circular concrete-filled steel tubulars (CCFST) members using piezoelectric lead zirconate titanate (PZT) patches.

Based on the previous studies performed by Xu et al. [10,11], the wave propagation in rectangular and circular CFST are different. The detailed wave propagation process and wavefield vary with the geometrical shape of CFSTs. In this study, the detectability of aggregate segregation in circular CFST components is numerically studied. In order to detect aggregate segregation in the concrete core of a circular CFST (CCFST) member, a PZT patch is mounted on the outer surface of the steel tubular as an actuator, and a PZT patch is embedded in the concrete core as a sensor. The PZT actuator is excited by a sweep frequency signal, and then, the voltage response of the PZT sensor is recorded to investigate the influence of concrete segregation on the output signals of the PZT sensor. The basic concept of the proposed concrete core segregation detection approach using the stress wave measurement of embedded PZT sensors is presented in Figure 1.

For the purpose of mimicking concrete core aggregate segregation, the multi-scale numerical concrete modeling approach with the ability to construct concrete core at the meso level is introduced. The geometries of coarse and fine aggregates, mortar, and the ITZs are generated with the numerical concrete generation program developed by the authors. By controlling the distribution of coarse and fine aggregates in the concrete core, the concrete core aggregate segregation at different levels can be simulated. In segregated concrete core, the aggregates are supposed to settle down to the bottom of the cross-section of the CFST member, and the mortar is located on the top of the cross-section, as shown in Figure 1.

### 2.1. Modeling of Segregated Concrete Core in CFST

In order to consider the effect of concrete core aggregate segregation on stress wave propagation and the response of embedded PZT sensor in CFST, a numerical concrete generation program is developed on the platform of MATLAB, and it is employed to generate concrete core with different aggregate distributions. In addition, the ITZ is also taken into account. The ideal aggregate gradation curve is employed to determine the size and quantity of different aggregates of the corresponding numerical model of a fully-graded concrete core [27]. Equation (1) describes the ideal aggregation curve:

(1) $$P=100\%\times \sqrt{\frac{D}{D_{\max}}}$$

where *P* is the percentage of aggregates that can pass through a mesh hole with the diameter of *D*, and $D_{\max}$ represents the maximum diameter of the characteristic aggregate.

A three-dimensional (3D) numerical concrete model at the meso scale is usually complicated, and the corresponding numerical simulation on stress wave propagation is extremely time-consuming. For simplicity, the Walraven method is adopted to simplify the aforementioned Fuller’s gradation curves to establish a two-dimensional (2D) planar model [28]. The volume percentage occupied by aggregates with a diameter less than *D*_0_, *P*(*D* < *D*_0_), is determined according to the following Equation (2):

(2) $$\begin{array}{cc} P(D<D_{0}) & =P_{k}[1.065(\frac{D_{0}}{D_{\max}}){}^{0.5}-0.053(\frac{D_{0}}{D_{\max}}){}^{4}-0.012(\frac{D_{0}}{D_{\max}}){}^{6}\\ & -0.0045(\frac{D_{0}}{D_{\max}}){}^{8}+0.0025(\frac{D_{0}}{D_{\max}}){}^{10}] \end{array}$$

where $P_{k}$ is the volume percentage of characteristic aggregates, and $D_{0}$ corresponds to the diameter of the sieve pore, respectively.

The numerical concrete sample generating procedure has been reported in detail [13]. The program for the generation of random aggregates in concrete cubes is similar to that for CFST structures, which is excluded herein.

**Figure 2:**
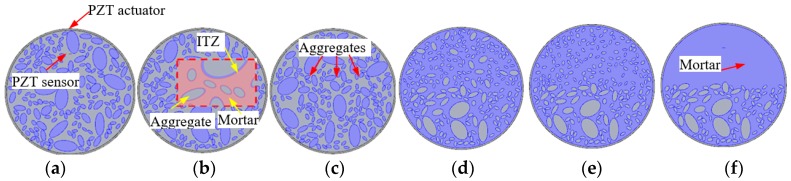
Circular concrete-filled steel tubulars (CCFST) meso-scale models with random numerical aggregates and segregation. (**a**) Sample 1; (**b**) Sample 2; (**c**) Sample 3; (**d**) Coarse aggregates segregation; (**e**) Coarse and middle aggregates segregation; (**f**) All aggregates segregation.

The outside diameter of the cross-section of the CCFST specimen to be studied herein is 400 mm, and the thickness of the circular steel tubular is 5.0 mm. The outline of the concrete core is defined as the packing boundary for three-graded random aggregates. The characteristic diameters of the three-graded aggregates employed to model the numerical concrete are 60 mm, 30 mm, and 15 mm, respectively, which represent the coarse aggregates (40~80 mm), middle aggregates (20~40 mm), and fine aggregates (5~20 mm). The input datum to the random aggregates generation and packing program include the aggregate gradation, ITZ thickness, and the packing boundaries. Based on the equations and parameters discussed above, the numerical concrete models with different aggregates distribution are generated as shown in Figure 2. Figure 2a–c presents the numerical concrete models with normally distributed aggregates, but without aggregate segregation, which are used to investigate the effect of the randomness of aggregates distribution on stress wave propagation. Figure 2d–f provides three numerical concrete core models with different aggregate segregation scenarios, which are adopted to analyze the effect of various levels of aggregates segregation on the stress wave propagation and the response of the embedded PZT sensor in the concrete core. Figure 2d represents a coarse aggregates segregation scenario. Figure 2e shows a scenario with both coarse and middle aggregates segregation. Figure 2f shows the scenario with all aggregates segregation.

### 2.2. Material Parameters of Multi-Scale Numerical Concrete

In this study, the concrete core of the CCFST member is decomposed of coarse and fine aggregates, mortar, and the ITZ. In the numerical simulation on the ultrasonic stress wave propagation excited by the surface-mounted PZT patch, the elastic material properties of each meso-scale component of the concrete core and steel tubular are shown in Table 1 [29,30]. The material parameters of piezoelectric ceramics are identical to those employed in the previous numerical studies [11].

### 2.3. Multi-Scale Multi-Physical Fields Coupling Model Composed of CCFST and PZT Patches

In order to consider the coupling effects between PZT patches and CCFST members, the multi-physical fields coupling model is established by sharing the finite element nodes between the CCFST member and the surface-mounted or embedded PZT patches while considering the piezoelectric effect of the PZT actuator and sensor. The surface-mounted PZT actuator is coupled with the steel tubular, and the embedded PZT sensor is coupled with the multi-scale numerical concrete core. The stress wave induced by the vibration of the surface mounted PZT actuator propagates through the cross-section of the CCFST member with the multi-scale concrete core model, and the electrical voltage response signal of the embedded PZT sensor subjected to the stress wave along its polarization direction is determined.

After establishing the multi-scale models of CCFST based on the random aggregate generation program, finite element meshing is performed with the multi-physical analysis software COMSOL as a solver. The dynamic equation of piezoelectric ceramics and the circuit state equation are described in Equations (3) and (4) [11,13,31]:

(3) $$\left[M\right]\left\{\ddot{u}\right\}+\left[K\right]\left\{u\right\}=V\left\{P\right\}+\left\{F\right\}$$

(4) $${\left\{P\right\}}^{T}\left\{u\right\}+C_{0}V=Q$$

where [*M*] is the general mass matrix, [*K*] stands for the stiffness matrix, {*F*} represents the load vector, *V* and *Q* are the electric potential on the electrode surface and the electricity of free charge, *C*_0_ is the clamp fixed capacitors, {*P*} is the electromechanical coupling vector, and {*u*} denotes the system displacement vector. Equation (5) shows the governing equations of the piezoelectric coupling system in the form of the generalized matrices and vectors.

(5) $$\left[\begin{array}{cc} \left[M\right] & \left[0\right]\\ \left[0\right] & \left[0\right] \end{array}\right]\{\begin{array}{c} \{\ddot{\mu }\}\\ \{\ddot{V}\} \end{array}\}+\left[\begin{array}{cc} \left[C_{d}\right] & \left[0\right]\\ \left[0\right] & \left[0\right] \end{array}\right]\{\begin{array}{c} \{\dot{\mu }\}\\ \{\dot{V}\} \end{array}\}+\left[\begin{array}{cc} \left[K\right] & \left[K^{Z}\right]\\ {\left[K^{Z}\right]}^{T} & \left[K^{d}\right] \end{array}\right]\{\begin{array}{c} \{\mu \}\\ \{V\} \end{array}\}=\{\begin{array}{c} \{F\}\\ \{Q\} \end{array}\}$$

where [*C_d_*] is the damping matrix, [*K^d^*] stands for the dielectric matrix components, and [*K^z^*] is the electromechanical coupling matrix components. When the PZT patch is employed as an actuator, {*F*} = {0} and {*Q*} = {*Q*(*t*)}. Similarly, when the PZT patch is employed as a sensor, {*F*} = {*F*(*t*)} and {*Q*} = {0} [11,31].

In the numerical simulation on the stress wave propagation in the cross-section of CCFST members, the maximum finite element size and the integration time step should be determined reasonably according to the minimum wavelength and the maximum frequency employed for segregation detection [10]. Ten to 12 degree of freedom(DOF) in per wavelength are better to describe the waveform, and Equation (6), which describes the relationship between the maximum dimension of element and the wavelength, should be satisfied when using second-order elements. In addition, the Courant–Friedrichs–Lewy (CFL) takes the values of 0.2 to achieve moderate accuracy [12]. Combined with Equation (6), the maximum integration time step can be obtained, and the mathematical expression is shown in Equation (7).

(6) h≤λ/5

(7) $$dt=CFL\times h/\max(c_{s},c_{P})=\min(T_{s},T_{P})/25$$

where *h* and λ correspond to the maximum element size and wavelength. The *c_s_*, *c_p_*, *T_s_*, and *T_p_* stand for the velocities of shear wave and the longitudinal wave and the corresponding time durations, respectively.

**Figure 3:**
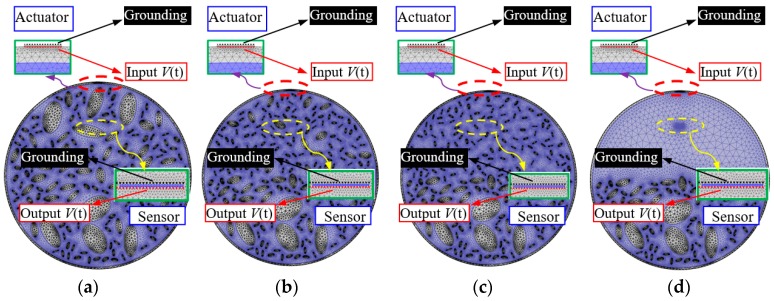
Meshing scheme of the piezoelectric lead zirconate titanate–circular concrete-filled steel tubulars (PZT-CCFST) coupling model (for 20 kHz) (**a**) without segregation; (**b**) coarse aggregates segregation; (**c**) coarse and middle aggregates segregation; (**d**) all aggregates segregation.

However, Equations (6) and (7) are the basic requirements for mesh size and integration time step. As shown in Figure 2b, fine meshing is needed to model the ITZ between aggregates and mortar at the meso scale. The minimum element size is relatively smaller than the predefined element size h. Figure 3 presents mesh examples of PZT-CCFST coupling models without and with aggregate segregation when an excitation frequency of 20 kHz is used. Figure 3a shows the meshing scheme of a PZT-CCFST coupling model with normally distributed aggregates. Figure 3b–d are the meshing examples of the coupling models with aggregate segregation at different levels (i.e., without segregation, coarse, coarse and middle, all aggregates segregation). The detailed material properties of the employed PZT patch, the definition of an electronic boundary, and Rayleigh damping are identical to that in the previous study performed by Xu et al. [11].

## 3. Multi-Scale Simulation on the Stress Wave Fields in CCFST

### 3.1. Stress Wave Propagation and the Wave Fields in CCFST with Normally Distributed Aggregates

In this section, the stress wave propagation process in the cross-section of CCFST excited by the PZT actuator is investigated. As mentioned above, the multi-physical coupling models at the meso scale are composed of aggregates, ITZ, mortar, steel plates, and PZT patches. In order to reduce the computational cost, a horizontal pulse force signal is applied at the outer surface of the steel plate to simulate the vibration of the PZT actuator, since the planner dimension of the PZT patch is much smaller when compared with the whole cross-section of the CCFST and the high linearity properties of the PZT material. The pulse force signal adopted in this section is described in Equation (8):

(8) $$F(t)=\{\begin{array}{cc} F_{0}\sin(2\pi ft){\left(\sin\left(\frac{2\pi ft}{5}\right)\right)}^{2} & t<\frac{5}{2f}\\ 0 & \mathrm{others} \end{array}$$

where *F*(*t*) and *F*_0_ correspond to the pulse force signal and its amplitude, *f* is the signal frequency, and *t* stands for the time variable in seconds. Here, *F*_0_ is set to 10^−7^ N, and the signal frequency *f* takes the value of 100 kHz.

**Figure 4:**
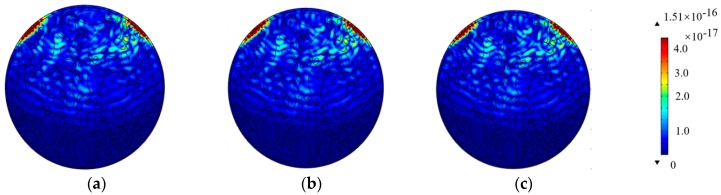
Comparison of the wave fields simulated with different mesh sizes (**a**) mesh size: 1.2 cm; (**b**) mesh size: 2.4 cm; (**c**) mesh size: 4.8 cm.

**Figure 5:**
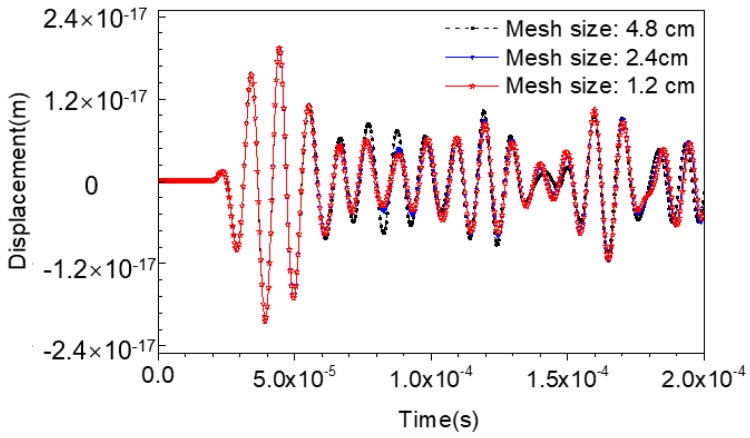
Mesh sensitivity analysis.

According to the material properties listed in Table 1 and Equations (6) and (7), the maximum element size should be limited to 2.4 cm. However, the wave propagation analysis based on finite element method (FEM) is sensitive to meshing size and integration time steps. In order to investigate the mesh independence in this study, numerical models with the maximum element sizes of 1.2 cm, 2.4 cm, and 4.8 cm are established accordingly. The stress wave fields and the time-history displacement curves at the location of the PZT sensor are presented in Figure 4 and Figure 5, respectively.

Figure 4 indicates that no significant difference can be observed in the stress wave propagation process in CCFSTs modeled with different mesh sizes, presenting similar waveform and wavefronts. Comparatively, the displacement-time curves can explicitly exhibit the waveform variation caused by mesh size. As presented in Figure 5, the waveforms of the head wave displacement at the location of the embedded PZT sensor, which is 10 cm away from the PZT actuator mounted on the surface of the steel tubular of the CCFST member, are very close. The whole waveforms calculated with the maximum element size of 1.2 cm and 2.4 cm are quite similar. However, the waveform corresponding to the model with the maximum mesh size of 4.8 cm shows deviations from the other curves. The results validate the reasonableness of the element size and the integration of the time step in this study.

**Figure 6:**
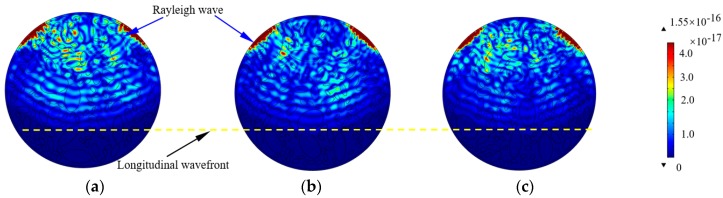
Comparison of the stress wave fields considering the aggregates distribution. (**a**) Elliptical sample 1; (**b**) Elliptical sample 2; (**c**) Elliptical sample 3.

Three different CCFST members with normally distributed aggregates are constructed to further discuss the effect caused by the random distribution of aggregates. Figure 6 shows the stress wave fields in CCFST models constructed with fully-graded elliptical aggregates in different distribution patterns at the time instant of 5.0 × 10^−5^ s. As shown in Figure 6a–c, the wavefronts of the longitudinal and shear waves become relatively blurred in the meso-scale numerical concrete cores compared with the stress wave propagation in homogeneous concrete [11]. Moreover, the wave amplitude attenuation can be detected to a certain extent due to reflection wave at the interfaces between mortar and aggregates, resulting in additional energy dissipation. However, there is no obvious difference between the longitudinal wavefronts presented in Figure 6a–c, which means that the velocity of stress wave is not significantly affected by the distribution variation of the elliptical aggregates with the same gradation. However, the crests of the shear wave are not clear due to the influence of interfacial reflections. Here, the wave fields are denoted by the magnitude of the total displacement of element nodes with the unit of meter.

### 3.2. Meso-Scale Stress Wave Propagation in CCFST with Aggregates Segregation

**Figure 7:**
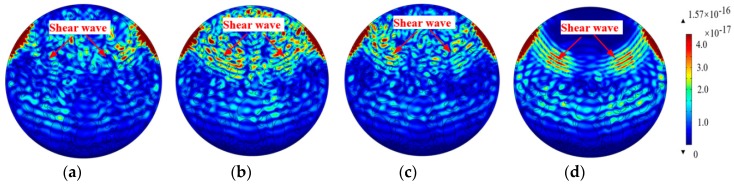
Comparison of the stress wave fields considering the aggregates segregation. (**a**) Without segregation; (**b**) Coarse aggregates segregation; (**c**) Coarse and middle aggregates segregation; (**d**) All aggregates segregation.

As discussed above, the effect of the random distribution of elliptical aggregates on stress wave propagation in the CCFST with the identical quantity and gradation of aggregates is limited. In engineering practice, aggregates may not be uniformly distributed during the casting of the concrete core, resulting in concrete aggregate segregation. Unlike the three samples with randomly distributed aggregates as presented in Figure 6, the effect of concrete aggregation on the stress wave propagation is studied with different aggregates segregation scenarios, as shown in Figure 7b–d. Figure 7b–d correspond to the coarse, coarse and middle, and all aggregates segregation scenarios, respectively. Figure 7a presents the stress wave of a CCFST member, where numerical concrete has normally distributed aggregates for comparison.

It can be observed from Figure 7 that the stress wave propagation is obviously affected by aggregates segregation. The longitudinal stress wave reaches the opposite border of the CCFST member without aggregates segregation, as shown in Figure 7a, at the time instant of 9.8 × 10^−5^ s. At the same time instant, the wave fields of the other three CCFST members with different aggregates segregation scenarios are shown in Figure 7b–d. It is clear that the wavefronts of the three CCFST members have slightly decayed due to aggregates segregation. Moreover, in the CCFTs member with all aggregates segregation, as shown in Figure 7d, the time delay of the head wave becomes more obvious. The shear waves can be clearly observed with stronger amplitude in the region filled with homogeneous mortar due to the disappearance of reflection waves between aggregates and mortar.

### 3.3. Effect of ITZ on Stress Wave Propagation in CCFST

**Figure 8:**
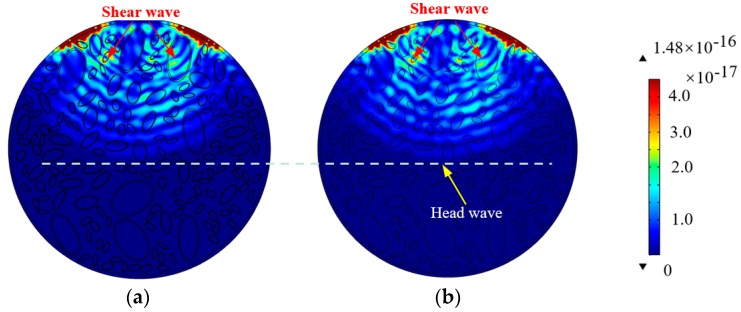
Comparison of stress wave fields considering the effect of the interfacial transition zone (ITZ). (**a**) Without ITZ; (**b**) With ITZ.

The ITZ plays critical roles in the mechanical behavior of concrete materials. Here, further investigation on the effect of IZT on the stress wave propagation of CCFST members is carried out with the help of numerical concrete modeling technology at the meso scale. The stress wave field snapshots corresponding to the CCFST with normally distributed aggregates with and without ITZ are presented in Figure 8a,b, respectively.

In this study, each ITZ is located at the outer of the corresponding aggregate and has the shape of an ellipse, and its long and short axes are 1.1 times those of its corresponding aggregates, respectively. However, the stress wave field and propagation pattern of the two models with and without the consideration of IZT are almost identical. Hence, numerical simulations show that the influence of the IZT on the stress wave propagation is very limited. It is reasonable to omit ITZ in the finite element (FE) modeling of CCFST members for stress wave propagation numerical simulation analysis for the purpose of improving computation efficiency.

It can be seen from Figure 6 to Figure 8 that the aggregates segregation of the concrete core affects stress wave propagation in CCFSTs. The effect caused by the randomness of meso-scale concrete core with normally distributed aggregates on the stress wave propagation is not dominant when compared with that of aggregates segregation. The numerical findings based on multi-scale simulation imply that aggregates segregation in the concrete core of CCFST members is detectable by using the stress wave measurement from PZT sensors embedded in the concrete core. In the following section, the response of the embedded PZT sensor under sweep frequency excitations is simulated and discussed in detail. An evaluation index called the normalized wavelet packet energy value (NWPEV) corresponding to the voltage measurement of the embedded PZT sensor is employed to assess the effect of aggregates segregation on the concrete core [12].

## 4. Sensitivity of Wavelet Packet Energy of Embedded PZT Measurement on Aggregates Segregation

The previous studies performed by the authors show that the PZT sensor measurement embedded in CFST members excited with a sweep frequency signal is sensitive to the interface debonding defect due to its wide frequency band [10]. Hence, in this study, the sweep frequency voltage signal is also employed to excite the PZT actuator mounted on the surface of the steel tubular of the PZT-CCFST coupling system, and the function expression is described as follows:

(9) $$V(t)=V_{0}\sin\left[2\pi f(t)t\right]$$

(10) $$f(t)=f_{0}+\frac{f_{1}-f_{0}}{T}t$$

where *V*(*t*) and *V*_0_ correspond to voltage excitation and its amplitude, and *f*(*t*), *f*_0_, and *f*_1_ stand for the frequency of the voltage signal at the time instant *t*, the initial frequency, and final frequency, respectively. Here, *V*_0_ takes the value of 10 V, and the time duration *T* equals to 0.001 s. *f*_0_ and *f*_1_ are set to 20 kHz and 40 kHz, respectively.

Numerical simulation on the stress wave propagation and the response of the embedded PZT sensor is carried out with the PZT-CCFST coupling models, considering the piezoelectric effect of PZT materials and the coupling effects between PZT patches and the CCFST member.

### 4.1. Damage Index Based on Wavelet Packet Analysis

For comparison, wavelet packet analysis on the output voltage signal of the embedded PZT sensor in CCFST members with a concrete core modeled with different aggregates segregation is carried out, and the corresponding wavelet packet energies are determined to describe the effect of aggregate segregation in this section.

**Figure 9:**
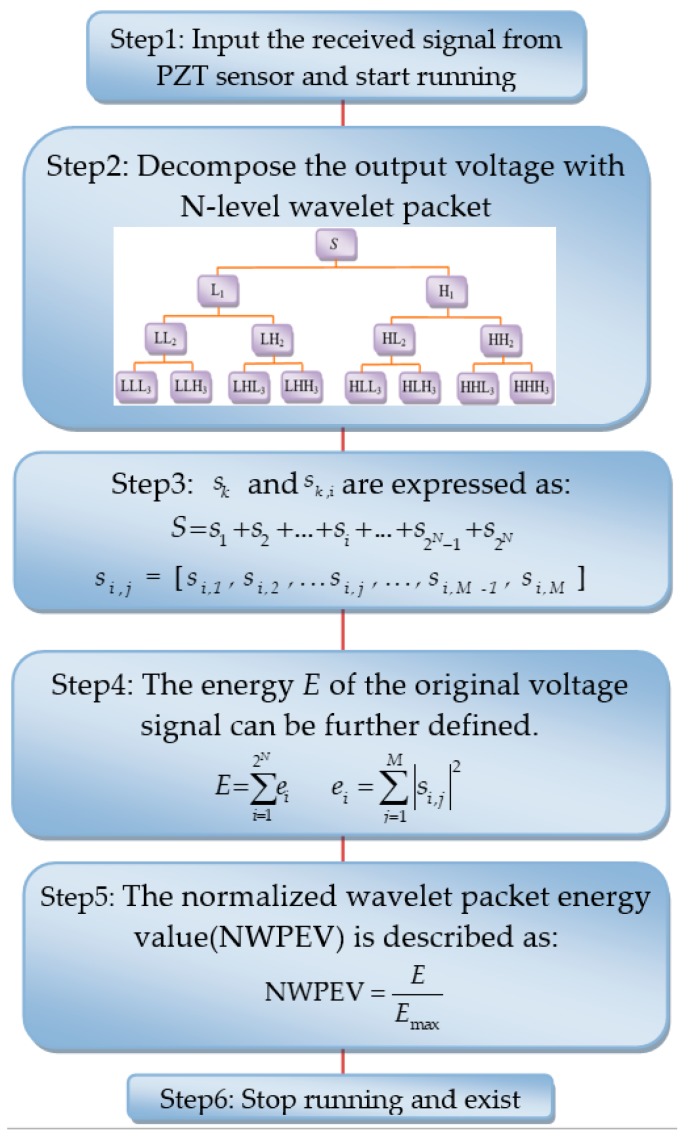
The flowchart of the determination of the normalized wavelet packet energy value (NWPEV).

Each original voltage signal of a PZT sensor can be expressed as a summation of 2*^N^* signal sets $s_{i,j}$ after decomposing by an *N*-level wavelet packet [32,33]. Then, a normalized wavelet packet energy value (NWPEV) is defined as an evaluation index. The detailed flowchart of the determination of NWPEV is presented in Figure 9, where *I* = 1, 2, ..., 2*N*, *j* = 1, 2, ..., *M. M* stands for the number of samples within the frequency band *k. E* is the wavelet packet energy value of voltage signals from the PZT sensor embedded in the concrete core with different aggregates distribution and segregation. *E*_max_ stands for the wavelet packet energy of the voltage signal of the PZT sensor corresponding to the concrete core established with three-graded random aggregates, which is taken as the reference value in the comparative analysis. In this study, *N* is set to 3.

### 4.2. Sensitivity on Normal Aggregates Distribution

**Figure 10:**
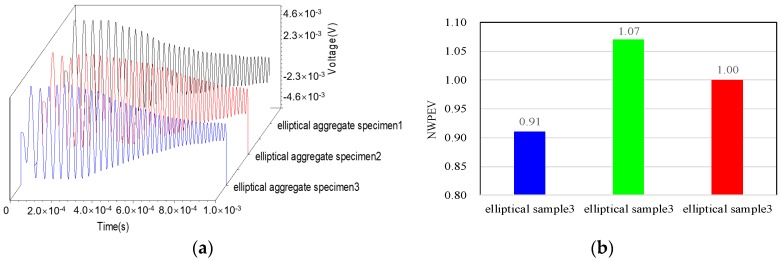
Output voltage and NWPEVs with different aggregate distribution. (**a**) Time–history curves of the output voltage; (**b**) Normalized wavelet packet energy value.

Before investigating the effect of aggregates segregation in the concrete core on the voltage response of the PZT sensor embedded in CFST members, the dynamic response of the PZT sensor in three CCFST members with fully-graded and normally distributed aggregates is analyzed first. Figure 10a shows the comparison of the time–history curves of the output voltage of the embedded PZT sensors in the CCFST members presented in Figure 2a–c. It can be seen that the voltage responses corresponding to different numerical concrete models with normally distributed aggregates are similar. The evaluation indices of NWPEV corresponding to three specimens are shown in Figure 10b, and the maximum error caused by the variation of aggregate distribution is less than 10.0%. The results meet well with the findings presented in Figure 6. The random distribution of aggregates poses only a local effect on the stress wave propagation in the concrete core.

### 4.3. Sensitivity on Aggregate Segregation

For the purpose of assessing the influence of aggregates segregation on the evaluation index, the voltage response of the embedded PZT sensor under the sweep frequency excitation is simulated. Here, the three numerical concrete models with different aggregates segregation scenarios shown in Figure 2d–f are taken as numerical examples, and the output voltage signals are compared with that of the CCFST member without aggregates segregation, as shown in Figure 2a.

**Figure 11:**
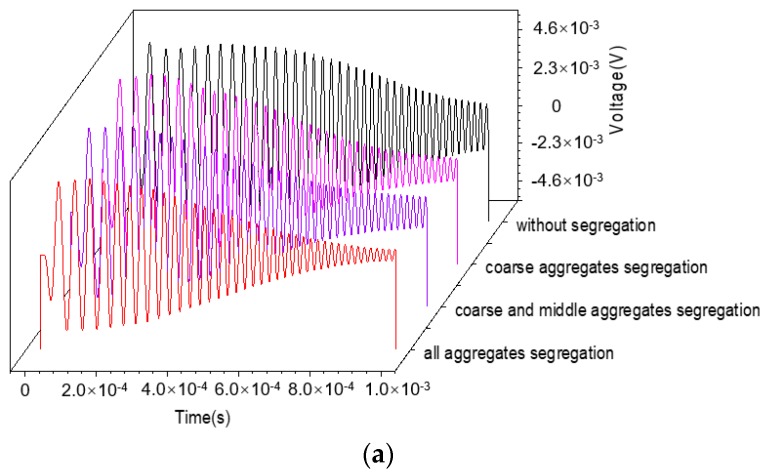

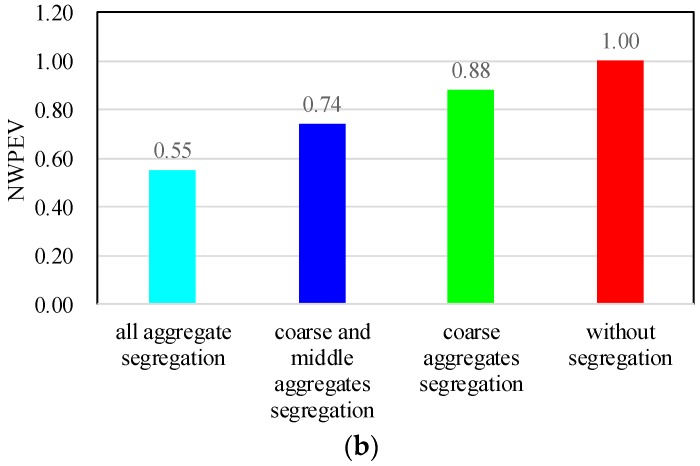
Output voltage and NWPEVs corresponding to aggregate segregation at different levels. (**a**) Time–history curves of the output voltage; (**b**) Normalized wavelet packet energy value.

Figure 11 presents the time–history curves of output voltage signals from the embedded PZT sensors of CCFST members with different aggregate segregation and the corresponding NWPEVs. The time history of the voltage response and the corresponding NWPEV of the embedded PZT sensor are sensitive to aggregates segregation. The evaluation index continuously reduces along with the increment of aggregates segregation levels. It can be concluded that the concrete aggregates segregation, which is a typical early defect in CFST structures, can be detected using the proposed stress wave propagation technique using PZT patches.

### 4.4. The Influence of the Presence of ITZ

**Figure 12:**
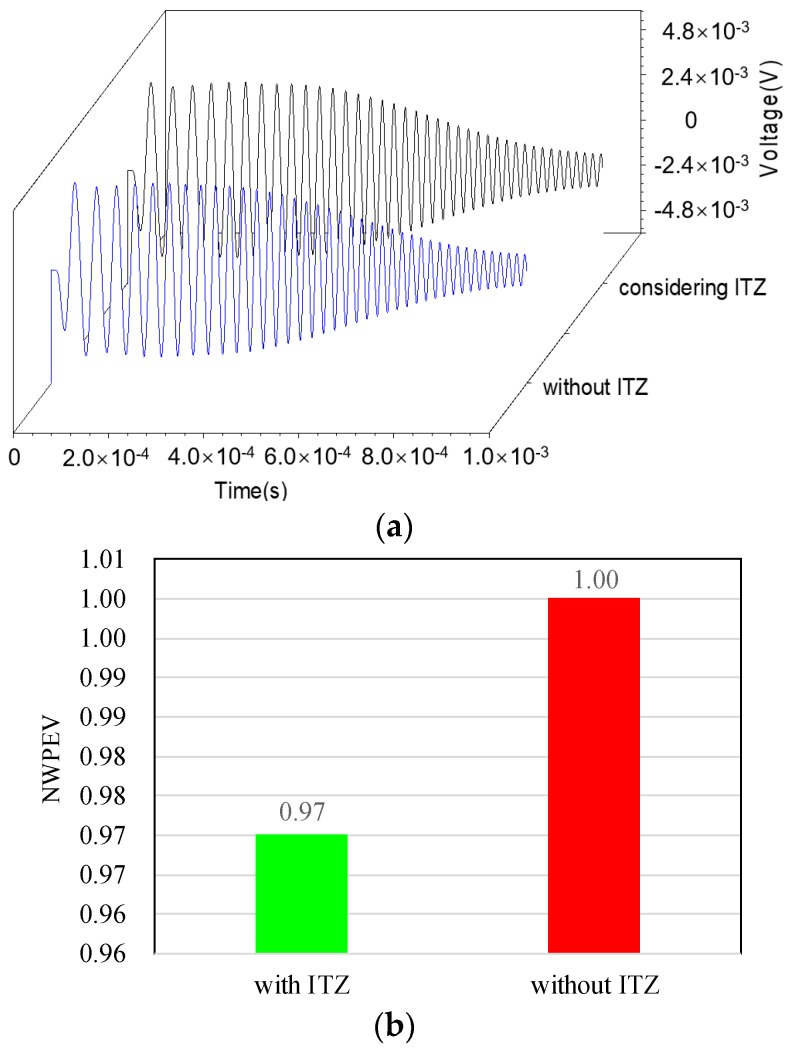
Output voltage and NWPEVs with and without considering ITZ. (**a**) Time–history curves of the output voltage; (**b**) Normalized wavelet packet energy value.

In this section, the difference in the output voltage of the PZT sensor embedded in numerical concrete core models with and without considering the ITZ is investigated. Figure 12 shows the comparison of the time–history output voltage of the embedded PZT sensors in the concrete core and the corresponding NWPEVs. As presented in Figure 12, the effect of the ITZ on the voltage response of the embedded PZT sensor is very limited. Even though the existence of ITZ leads to additional reflection waves between aggregate and the mortar, the corresponding energy loss is not obvious, since the thickness of the ITZ is relatively small. Therefore, the influence of ITZ on the stress wave propagation and response of the PZT sensor of the PZT-CCFST coupling model can be ignored in the multi-physics and multi-scale simulation.

## 5. Conclusions

In this study, for the purpose of investigating the detectability of aggregates segregation in the inaccessible concrete core of CCFST members, multi-physical and multi-scale coupling numerical models composed of a surface-mounted PZT actuator, a CCFST component with a meso-scale numerical concrete core, and an embedded PZT sensor are established at first. With the help of random aggregates generation and a packing program, a concrete core is decomposed as the combination of three-graded elliptical aggregates, mortar, and the ITZ. The stress wave propagation process and the stress wave fields are comparatively discussed. The influence of meso-scale structure variation in the concrete core on the stress wave fields and the output voltage of the embedded PZT sensor are investigated in detail. The major conclusions can be made as follows:

The numerical concrete modeling approach based on random aggregate generation and the packing method is employed to model the meso-scale structure of the concrete core in CCFST members, which is composed of elliptical aggregates with different dimensions and distribution, mortar, the ITZ. The numerical concrete modeling approach provides an efficient way to investigate the effect of meso-scale structure variation of the concrete core and aggregates segregation in the concrete core on the stress wave propagation as well as the voltage response of embedded PZT sensor measurement in the cross-section of CCFST members.

The initiation and propagation process of stress waves in CCFSTs modeled with fully-graded random aggregate samples with normal distribution are investigated at first via the multi-scale and multi-physical fields coupling FE models. The results indicate that the stress wave velocity is not significantly affected while changing the distribution pattern of three-graded elliptical aggregates samples without aggregates segregation. Besides, the existence of ITZ also poses a limited influence on stress wave propagation. However, the stress wave fields are obviously influenced by the aggregates segregation of the concrete core in CCFST members.

The time–history voltage signal of the embedded PZT sensor and the corresponding evaluation index NWPEV based on wavelet packet analysis are sensitive to the aggregates segregation of the concrete core of CCFST members. The evaluation index NWPEV consistently drops with the level increment of aggregates segregation. In contrast to aggregates segregation, the influence of the existence of ITZ and the randomness distribution of fully-graded aggregates without segregation is not obvious. The effect of aggregates segregation on the stress wave propagation in the cross-section of CCFST members and the time–history voltage response of embedded PZT sensor is dominant.

The findings imply that aggregates segregation, as a typical early age defect in the concrete core of CCFST members, can be identified efficiently by using the stress wave propagation measurement from PZT sensors. The early age aggregate segregation detection is very important and meaningful, because countermeasures can be carried out to handle the defect before the hardening of the concrete core. The feasibility of the proposed PZT-based concrete aggregate segregation detection approach for CCFST members is verified numerically in this study by taking advantage of the convenience of modeling the aggregates segregation in numerical concrete models. However, based on the findings from the numerical simulation results in this study, experimental study on the feasibility of the proposed aggregates segregation detection technique for CCFST members can be carried out in the follow-up studies, where the preparation of the concrete core in CCFST members with desired aggregates segregation scenarios will be a challenging task. In order to model the real distribution of aggregates in concrete, scanning electron microscopy (SEM) photographs technology is helpful.

## Figures and Tables

**Table 1:** Material parameters of meso-scale concrete core and steel tubular [29,30].

Material	Young’s Modulus (GPa)	Poisson’s Ratio	Density (kg/m^3^)
Aggregates	55.5	0.16	2700
Mortar	26.0	0.22	2100
ITZ	25.0	0.16	2400
Steel	207.0	0.280	7800
